# Long-Term Survival of Patients with Relapsed/Refractory Acute Leukemia Receiving Clofarabine/Cyclophosphamide Prior to Hematopoietic Stem Cell Transplantation

**DOI:** 10.3390/cancers18213593

**Published:** 2026-11-09

**Authors:** Irene Graf, Philipp Wohlfarth, Michael Kundi, Margit Mitterbauer, Axel Schulenburg, Alexander W. Hauswirth, Karoline V. Gleixner, Hanna A. Knaus, Lina Z. Rüsing, Werner Rabitsch, Peter Valent, Wolfgang R. Sperr

**Affiliations:** 1 Division of Hematology and Hemostaseology, Department of Internal Medicine I, Medical University of Vienna, 1090 Vienna, Austria; irene.graf@meduniwien.ac.at (I.G.);; 2 Ludwig Boltzmann Institute for Hematology and Oncology, Medical University of Vienna, 1090 Vienna, Austria; 3 Bone Marrow Transplantation Unit, Department of Internal Medicine I, Medical University of Vienna, 1090 Vienna, Austria; 4 Center for Public Health, Department of Environmental Health, Medical University of Vienna, 1090 Vienna, Austria

**Keywords:** AML, ALL, refractory leukemia, relapsed/refractory leukemia, hematopoietic stem cell transplantation, bridging therapy, clofarabine, cyclophosphamide

## Abstract

Background: Allogeneic hematopoietic stem cell transplantation (HSCT) is the only potentially curative option for patients with drug-refractory acute myeloid (AML) or acute lymphoblastic leukemia (ALL). Patients and Methods: We retrospectively analyzed 82 patients (median age 47.5 years, range 19–69; female:male ratio 1:1.28) with drug-refractory (R) or relapsed/refractory (RR) acute leukemia (AML *n* = 66; ALL *n* = 16) who were treated with clofarabine (10 mg/m^2^) and cyclophosphamide (200 mg/m^2^) days 1–4 (ClofCy) prior to HSCT. Results: ClofCy resulted in rapid cytoreduction (median time to granulocytes <0.5 × 10^9^/L: 5 days). HSCT was performed in 62.2% (51/82) of our patients. Infections (18/31, 58%) were the main reason to withhold transplantation. HSCT and non-HSCT patients differed significantly regarding the median number of chemotherapies received prior to ClofCy (HSCT, median = two therapies; non-HSCT, median = three therapies; *p* = 0.027). Median overall survival (OS) was 0.31 years in all patients (5- and 10-year OS; 24% and 20%, respectively). HSCT patients had a median OS of 1.23 years (5- and 10-year OS; 38.4% and 31%, respectively) and those in the non-HSCT cohort a median OS of 0.11 years (5-year OS; 0%). Median OS and relapse-free survival (RFS) in the HSCT patient cohort were 1.04 and 0.9 years, respectively. Acute graft-versus-host disease (aGvHD) was associated with inferior survival (median OS 0.46 years), whereas chronic graft-versus-host disease (cGvHD) was associated with improved OS and RFS (above 50%). Conclusion: ClofCy is an effective bridging regimen enabling HSCT in patients with R/RR acute leukemia. Introducing ClofCy may improve the probability of reaching HSCT and long-term survival in this cohort of patients with poor prognosis.

## Simple Summary

Although treatment outcomes have improved substantially over the past few decades, most adult patients with acute leukemia do not have long-lasting responses to standard chemotherapy. For these patients, hematopoietic stem cell transplantation is the only potentially curative therapy option. However, in many of these cases, it is difficult to control the drug-resistant leukemia to safely undergo transplantation. We evaluated whether intensive cytoreduction with clofarabine and cyclophosphamide is an effective approach to shrink the leukemia burden in such patients before transplantation. Indeed, this therapy effectively reduced the disease burden and enabled most patients to proceed to transplantation, resulting in an improved overall survival. Our data show that stem cell transplantation may be an option even in highly refractory/resistant acute leukemia cases and that therapy with clofarabine and cyclophosphamide shortly before transplantation may result in more transplantable cases and more patients entering long-term complete remission following stem cell transplantation.

## 1. Introduction

Acute myeloid leukemia (AML) and acute lymphoblastic leukemia (ALL) are life-threatening stem cell malignancies that pose a significant challenge to our health care system [1,2,3,4]. Both malignancies are characterized by a rapid and uncontrolled growth and expansion of leukemic precursor cells in the bone marrow (bm), peripheral blood (pb), and other organs [1,2,3,4]. The prognosis and clinical course in AML and ALL depend on a multitude of disease-related factors, including leukemia subtype, cellular and histopathological features, as well as genetic and molecular abnormalities [1,2,3,4,5,6,7,8,9,10]. Patient-specific factors, such as age and the presence of pre-existing medical conditions (comorbidities) may also play a critical role in disease evolution and outcome [1,2,3,4,5,6,7,8,9,10].

The probability to achieve complete remission (CR) with best available chemotherapy ranges between 60% and 75% in AML and between 60% and 85% in ALL patients [1,3,9]. However, blast cell persistence is seen in 25–40% and 15–40% of the patients with AML and ALL, respectively. Moreover, a substantial number of patients experience a relapse after chemotherapy, whereas others present with blast cell persistence during therapy [1,2,3,4,9].

Patients with de novo acute leukemia who do not enter CR following two cycles of initial chemotherapy, referred to as refractory (R) disease, face an exceedingly poor prognosis. Similarly, patients experiencing a relapse of leukemia who do not achieve a second CR following salvage chemotherapy (RR) have a poor chance of survival [11,12,13,14,15,16]. For such patients, allogeneic hematopoietic stem cell transplantation (HSCT) represents the only potential curative treatment option [11,12,13,14].

Several studies have reported on the importance of chemotherapy to achieve CR or to reduce blast cell levels to less than 20% before HSCT [13,17,18,19]. Thus, repetitive cycles with multi-agent chemotherapy protocols, including FLAG-Ida (fludarabine, cytarabine, and idarubicin) [12] or high-dose cytarabine combined with mitoxantrone (HAM) [13], are used with the goal of achieving CR before HSCT. However, these therapies often result in poor CR rates [12,13]. Moreover, since CR is usually only achieved after repeated cycles of chemotherapy, there is a high risk of adverse events due to infections or bleeding [20,21]. Moreover, low graft-versus-host disease (GvHD) rates but high percentages of non-relapse mortality due to infections and other toxicities were observed, particularly in patients with compromised performance status [20]. The study by Shahswar et al. evaluated the safety of the FLAVIDA regimen (fludarabine, cytarabine, idarubicin, and venetoclax) and once again highlighted severe hematologic complications based in infections as a key concern [22]. Prolonged chemotherapy prior to HSCT raises concerns about heightened toxicity and the potential for a delay or ineligibility regarding HSCT due to prolonged severe infections, including systemic fungal infections [16].

In recent years, several studies have reported on rapid debulking strategies to reduce the number of malignant cells quickly (with or without CR) instead of induction with the aim to strictly achieve a CR. After debulking, patients proceed to conditioning therapy without waiting for hematologic recovery [15,17,18,22,23]. Clofarabine in combination with cyclophosphamide (ClofCy) to reduce the leukemic burden was firstly introduced by Karp et al. in 2007 for patients with R/RR acute leukemias [24,25]. Locke et al. demonstrated that clofarabine was effective for cytoreduction pre-HSCT with acceptable toxicities [17]. An overall survival (OS) of 71% in a small sample of 29 patients was achieved in this study [17]. We previously reported similar promising data using ClofCy for bridging chemotherapy refractory patients with newly diagnosed refractory or relapsed acute leukemias prior to HSCT [15]. In a small cohort of 20 patients, a median survival of 531 days post-HSCT was achieved. Six patients were found to be still alive after a median follow-up of 1245 days [15].

In this study, we report on the outcome of an extended cohort of patients that was bridged to HSCT using ClofCy and was followed for a longer time period extending up to 15 years. The specific aims of this project were to further define the role of ClofCy as an effective debulking strategy prior to HSCT in patients with R/RR acute leukemia, to evaluate long-term survival outcomes, and to identify predictive clinical factors influencing prognosis and outcome in this high-risk population.

## 2. Patients and Methods

In this study, we retrospectively analyzed patients with acute myeloid and lymphoid leukemias who underwent debulking chemotherapy with ClofCy prior to a planned conditioning therapy for HSCT at the Medical University of Vienna. ClofCy was used in case of R/RR leukemia with an available related or unrelated donor and absence of solid manifestations of leukemia. We retrospectively collected data on patients’ disease at diagnosis, the clinical status of the patients’ previous cytoreductive treatments, debulking therapy prior to HSCT, and outcome of HSCT by chart review. The study included all patients treated with ClofCy between November 2008 and September 2023 in our center. Details on patient selection, ClofCy treatment, conditioning, HSCT, and the reasons for not proceeding to HSCT are depicted in Figure S1. All analyses were conducted with approval from the local ethics committee of the Medical University of Vienna (EK 1676/2024).

### 2.1. Patients

Eighty-two patients (median age: 47.5 years; range: 19–69) diagnosed with R/RR acute leukemia received debulking therapy with ClofCy. Among these, 66 had AML (80.5%; median age: 48 years; range: 20–69) and 16 ALL (19.5%; median age: 37 years; range: 19–61). Forty-eight patients (58.5%) had refractory disease following first-line therapy, and 34 (41.5%) had experienced a refractory relapse. There were 36 female (median age: 45 years; range: 19–63) and 46 male patients (median age: 50.5 years; range: 20–69; f:m ratio: 1:1.3). Detailed patients’ characteristics are shown in Table 1.

### 2.2. Therapy and Response Evaluation

Each cycle of ClofCy consisted of intravenous administration of clofarabine at a dose of 10 mg/m^2^ on days 1 to 4, along with cyclophosphamide at a dose of 200 mg/m^2^ on days 1 to 4 [15]. One to three cycles of ClofCy were planned, depending on the response to treatment and the time required to bridge until a suitable donor became available. In case of a delay of HSCT, more cycles of ClofCy could be given to bridge for HSCT. In patients with refractory disease to first-line therapy, ClofCy was applied after the second induction; in cases with relapsed disease, ClofCy was given after one cycle of salvage therapy. To assess the response to ClofCy, a bm aspirate and bm biopsy were performed both before initiating this therapy and, in most patients, bm was also examined after the final cycle of ClofCy.

### 2.3. Stratification of Patients and ClofCy-Related Toxicity

We used the Hematopoietic Cell Transplantation-Comorbidity Index (HCT-CI) to assess the severity of comorbidities, with severity grades ranging from 0 to 5, defining 0 as low risk, 1 to 2 as intermediate risk, and 3–4 as high risk, and 5 or more as very high risk. Toxicities were assessed according to the Common Terminology Criteria for Adverse Events (CTCAE), version [v4.0], which classifies adverse events into five grades: grade 1 (mild), grade 2 (moderate), grade 3 (severe), grade 4 (life-threatening), and grade 5 (death related to adverse event). Hepatic and renal toxicities were evaluated based on laboratory parameters, including serum levels of aspartate aminotransferase (AST), alanine aminotransferase (ALT), gamma-glutamyl transferase (GGT), alkaline phosphatase (AP), bilirubin, and creatinine. Grading was performed in accordance with CTCAE thresholds for each parameter to ensure standardized reporting of toxicity severity. These parameters were captured before initiation of ClofCy and either on day 0 of HSCT or 30 days after the end of ClofCy in non-HSCT patients.

### 2.4. HSCT and Evaluation of GvHD

Conditioning therapy was initiated at the time of aplasia following treatment with ClofCy. The type of conditioning regimen depended on age, physical performance, disease variant, transplantation protocol, and individual clinical considerations. Patients received either full dose (myeloablative) or intermediate dose conditioning therapy. Six different full dose, and three intermediate dose regimens were used. Details of conditioning regimens are depicted in Tables S1 and S2. Peripheral blood stem cells (PBSC) were used as stem cell source, except one case where cord blood stem cells were used. For patients undergoing myeloablative conditioning therapy, GvHD-prophylaxis consisted of cyclosporine A (CSA) with a target plasma level of 200–250 ng/mL and methotrexate (MTX) at a dose of 15 mg/m^2^ on day +1, followed by 10 mg/m^2^ on days +3, +6, and +11, following the Seattle protocol (CSA + MTX) [15]. For patients receiving intermediate dose (reduced) intensity conditioning, CSA and mycophenolate mofetil (MMF) at a dose of 15 mg/kg/day were administered from day 0 until complete donor cell chimerism was established [15]. Anti-thymocyte globulin (ATG) was used as GvHD prophylaxis alone or in combination with CSA + MMF (Table S3).

GvHD was classified according to the National Institutes of Health (NIH) consensus criteria [25]. Acute GvHD (aGvHD) included classic acute GvHD occurring within the first 100 days after HSCT, as well as persistent, recurrent, and late-onset acute GvHD presenting beyond day 100 in the absence of chronic GvHD (cGvHD) manifestations [25]. Chronic GvHD was diagnosed based on characteristic NIH-defined clinical features irrespective of the time after transplantation [25].

### 2.5. Supportive Care

Patients received red blood cell transfusions as needed to ensure hemoglobin levels above 8.0 g/dL. Platelet (PLT) transfusions were administered to maintain PLT counts above 10 G/L. In cases of severe infection or mucositis, PLT transfusions were given to maintain a PLT count of at least 20 G/L [16]. Additionally, all patients were provided with prophylactic measures such as cotrimoxazole for the prevention of Pneumocystis jirovecii infection and acyclovir for cytomegalovirus prophylaxis, in accordance with established protocols [24].

### 2.6. Statistical Analyses

For descriptive statistics, metric data are presented as median, inter-quartile range, categorical data as absolute and relative frequencies. OS was defined as time from start of therapy to death. Patients still at risk were censored. Relapse-free survival (RFS) was defined as time from achieving a complete remission (CR) to relapse or death. Patients still at risk were censored. The product limit method of Kaplan and Meier was used to analyze the probability of OS and RFS. Differences in survival among cohorts were assessed by log-rank test. Multivariate analyses were performed by Cox regression with and without time-dependent covariates. The effect of acute and chronic GvHD on survival was assessed by Cox regression with the Schoenfeld residuals test applied to assess time-dependency and to test the proportional hazard assumption. In addition, a landmark analysis using the product limit method of Kaplan and Meier with 100 days lag was applied. The relapse- and non-relapse mortality were analyzed using the Aalen–Johansen survival estimator. To evaluate the toxicity according to the number of ClofCy cycles the Jonckheere–Terpstra test was used. Patients with missing data were not included in the analyses and no imputation was applied. Differences were considered significant with a *p*-value < 0.05. Statistical calculations were carried out in Microsoft Excel for MAC Version 16.16, IBM SPSS Statistics for macOS Version 15.7.7 and Stata 17.

## 3. Results

### 3.1. Prognostication of Patients According to the HCT-CI

Regarding the HCT-CI 45 patients (54.9%) were in the low-risk group, 26 (31.7%) in the intermediate risk group, and 11 (13.4%) in the high-risk group, without significant differences between ALL and AML patients.

### 3.2. Efficacy of ClofCy

A median number of two cycles of ClofCy (range 1–5) was administered. In 22 patients, one cycle was given, 26 patients received two cycles, 21 patients three cycles, eleven patients four cycles and one patient received five cycles. Patients with AML received a median number of 2 (range 1–5) ClofCy cycles, and those with ALL a median number of 2.5 cycles of ClofCy (range 1–4) (Table S4). Therapy with ClofCy resulted in a significant cytoreduction with a median time to granulocytes < 0.5 × 10^9^/L of 5 days (interquartile range [IQR] 2.8–7 days). In the non-HSCT cohort the median time to white blood cells (WBC) below 0.5 × 10^9^/L was 4.5 days (range 0–8 days) and did not differ significantly from HSCT patients (*n* = 51, median age 44 years, IQR 35–57) with a median duration until WBC dropped below 0.5 × 10^9^/L of 5 days (ranged 0–28) (*p* = 0.99).

Paired bone marrow blast percentages before and after treatment with ClofCy were evaluated in 48 patients. A decrease in blast percentage was observed in the majority of patients (*n* = 36, 73.5%), while blasts remained unchanged in 16% (*n* = 7) and increased in 10% (*n* = 5). The corresponding data are shown in Figure S2 and Table S5.

### 3.3. Tolerability of ClofCy

In most patients, liver enzymes and serum creatinine levels (crea) did not increase following treatment with ClofCy (Table 2). Increasing AST, ALT, GGT, AP, bilirubin and crea were found in 15 (18.5%), 19 (23.4%), 22 (27.1%), 11 (13.5%), seven (8.7%), and nine (11.1%) patients, respectively (Table 2). The changes in liver and renal parameters as assessed by CTCAE (grade 1–4) following therapy with ClofCy, in HSCT and non-HSCT patients are shown in Table S6. In a majority of these patients, toxicities were grade 1–2 according to CTCAE. A grade 3–4 increase in AST and ALT was observed in two patients (2.4%). In five patients (6.1%) a grade 3–4 increase in crea was observed (Table 2). Four of these had multi-organ failure, i.e., two patients after the second and two after the third cycle of ClofCy. Three of these patients had CTCAE grade 3 or 4 toxicities (liver and kidney, *n* = 2; kidney, *n* = 1). To further assess toxicity according to the number of ClofCy cycles, the highest serum levels of ALT, AST, GGT, ALP, bilirubin, and creatinine during days 1–30 after HSCT were recorded. Patients were grouped according to the number of ClofCy cycles received (1, 2, 3, or >3 cycles) and compared using the Jonckheere–Terpstra test. No significant relationship between the number of cycles and liver toxicity or renal toxicity in any of the assessed laboratory parameters were observed (*p* > 0.05; Figure S3).

Thirty-one patients (37.8%) did not proceed to HSCT (median age 49 years, IQR 36–71). Severe infections with clinical deterioration (*n* = 19), progressive disease despite ClofCy (*n* = 4), fungal infections (*n* = 3) and intracranial hemorrhage (*n* = 3), were causes to withhold HSCT (Figure S1). Infections were the most frequent reason for not proceeding to HSCT and were associated with a significantly longer duration of antimicrobial therapy in the non-HSCT group than in the HSCT group (median: 20.5 vs. 10 days; *p* = 0.011; Figure S4). The number of blood cultures showing bacterial growth was significantly higher in the non-HSCT group (*p* = 0.009; Table S7). Intravenous antimicrobial therapy was administered in 38/51 (74.5%) HSCT patients and 26/31 (83.9%) non-HSCT patients, with significantly more modifications of antimicrobial therapy in the non-HSCT group (*p* = 0.011; Table S8).

Three patients in the non-HSCT group and five in the HSCT group required ICU admission during ClofCy therapy. No severe mucositis was documented prior to HSCT. Among HSCT patients, median neutrophil recovery occurred after 51.5 days (IQR: 39–60), with 5/51 patients (9.8%) failing to achieve full neutrophil recovery. Median platelet recovery occurred after 31 days (IQR: 25–39.5), with 9/51 patients (17.6%) failing to achieve full platelet recovery. The mortality rate of all patients at day 30 and 60 after the initiation of ClofCy was 17.1% and 34.4%, respectively.

### 3.4. Prognostic Factors for Successful Bridging to HSCT

The non-HSCT and HSCT cohort did not differ significantly regarding age (*p* = 0.33), type of disease (myeloid or lymphoid) (*p* = 0.585), or R versus RR disease (*p* = 0.392). The proportion of patients classified as high-risk according to the HCT-CI was markedly but not significantly higher in the non-HSCT group (24.1%) than in the HSCT group (8.3%) (*p* = 0.152). HSCT patients had fewer bm blasts after ClofCy compared to non-HSCT patients, with a tendency toward statistical significance (*p* = 0.059). The number of chemotherapies prior to ClofCy differed significantly between HSCT and non-HSCT patients (*p* = 0.027; Table 3). As assessed by multivariable analysis, including age, sex, type of leukemia and R versus RR leukemia, the number of previous chemotherapies prior to ClofCy was the only independent prognostic variable for patients to proceed to HSCT (*p* = 0.032; Table 3). Grade 3–4 toxicities according to CTCAE were only observed in patients not proceeding to HSCT.

### 3.5. Engraftment After HSCT and Rates of Acute and Chronic GvHD

Depending on age, physical performance, disease type, and individual clinical considerations patients received either full dose (myoablative) (*n* = 39) or reduced toxicity (*n* = 12) conditioning therapy. Detailed information on the regimens used is included in the Tables S1 and S2. HSCT was performed using PBSC from a matched related donor (*n* = 12, 23.5%), related haploidentical donor (*n* = 3, 5.9%), matched unrelated donor (*n* = 29, 56.9%), mismatch unrelated donor (*n* = 6, 11.7%), and one cord blood donor (2.0%). Transplant-related mortality at day 60 was 17.6%. The median time to an absolute neutrophil count (ANC) > 0.5 G/L (for three consecutive days) and PLT > 20 G/L (without transfusion) was 17 (IQR: 13–22) days and 14.5 (IQR: 11–40) days after HSCT, respectively. In 43 patients (84.3%) engraftment of ANC and PLT was observed. Three patients had no engraftment of PLT and in 5 patients, neither WBC nor PLT engrafted. Four of these 8 patients with engraftment failure had blast cell persistence.

Most patients with engraftment failure died from septicemia and multi-organ-failure (*n* = 5). Two patients had an early relapse. The causes of death are depicted in Table S9. In one patient the cause of death was not recorded. GvHD prophylaxis was performed according to protocol in all patients (Table S3). Ten patients developed aGvHD, nine within 60 days from transplantation and one after reduction in immunosuppression after that. A total of 11 patients developed chronic GvHD (cGvHD), including two with a prior history of acute GvHD. Normally, reduction in immunosuppression started around day 60, 22 patients received immunosuppression beyond day 100 due to GvHD.

### 3.6. Causes of Death

Overall, infections were the leading cause of death in our patients. Septicemia with multi-organ failure accounting for most cases (*n* = 21), predominantly in non-HSCT patients (*n* = 16, 87.5%) (Table S9). Infections leading to death included bacterial infections (*n* = 21), two aspergillus pneumonia infections, a candida infection, and one COVID-19 infection, all occurring in the non-HSCT group. Deaths after HSCT were less frequently recorded and were primarily related to septicemia (*n* = 5) and, in a few cases, related to refractory disease and/or acute graft-versus-host disease. Other non-infectious causes of death observed in non-transplanted patients, were clinical deterioration (*n* = 4), refractory disease (*n* = 5), and intracranial hemorrhage (*n* = 3).

### 3.7. Survival After HSCT: OS and RFS

**Figure 1:**
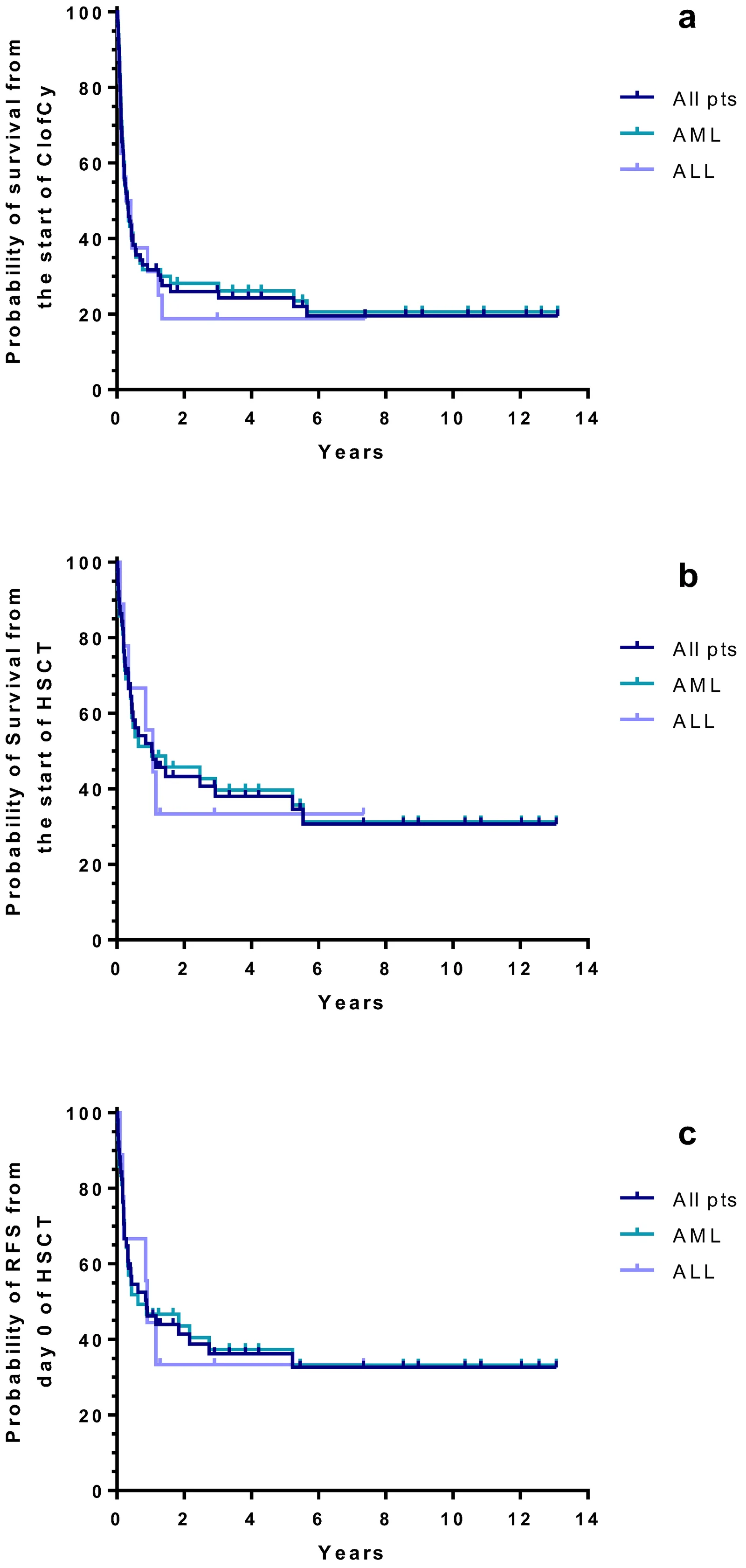
Survival of ClofCy patients. (**a**) Overall survival of all 82 patients from initiation of ClofCy as assessed by the Kaplan–Meier method. There was no significant difference between AML and ALL patients; (**b**) overall survival of patients who underwent HSCT from day one of HSCT; (**c**) relapse-free survival of patients who underwent HSCT from day one of HSCT. Again, there was no significant difference between AML and ALL patients. Abbreviations: ALL, acute lymphoblastic leukemia; AML, acute myeloid leukemia; ClofCy, clofarabine/cyclophosphamide; HSCT, hematopoietic stem cell transplantation; pts, patients.

With a median duration of observation of 7.38 years, the median OS from day one of ClofCy was 0.31 years (IQR 0.12–3.02) (Figure 1a). The 5- and 10-year survival rates were 24% and 20%, respectively. The median OS from day one of HSCT was 1.23 years with an IQR of 0.22-not reached (nr) and a 5- and 10-year survival of 38.4% and 31%, respectively (Figure 1b). The median RFS in these patients was 0.9 years (Figure 1c). RFS at 5 years was 37.3%. The median OS in those without HSCT was 0.11 years (IQR 0.06–0.17) and the 5-year survival 0%. Subgroup analyses according to treatment period (11/08–04/16 vs. 05/16–09/23), number of ClofCy cycles (1 cycle; 2 cycles; 3 cycles; >3 cycles), and donor type (matched sibling, haplo-identical sibling, matched unrelated, mismatched unrelated donor or cord blood) showed no significant differences in overall survival (*p* > 0.1; Figures S5–S7). To address selection and immortal-time bias in the comparison between HSCT and non-HSCT patients, we calculated the survival of our patients according to the Aalen-Johansen survival estimator with relapse as competing risk. As shown in Figure S8, we found no major difference compared to the survival calculated by the Kaplan–Meier method.

### 3.8. Prognostic Factors in HSCT Patients

**Figure 2:**
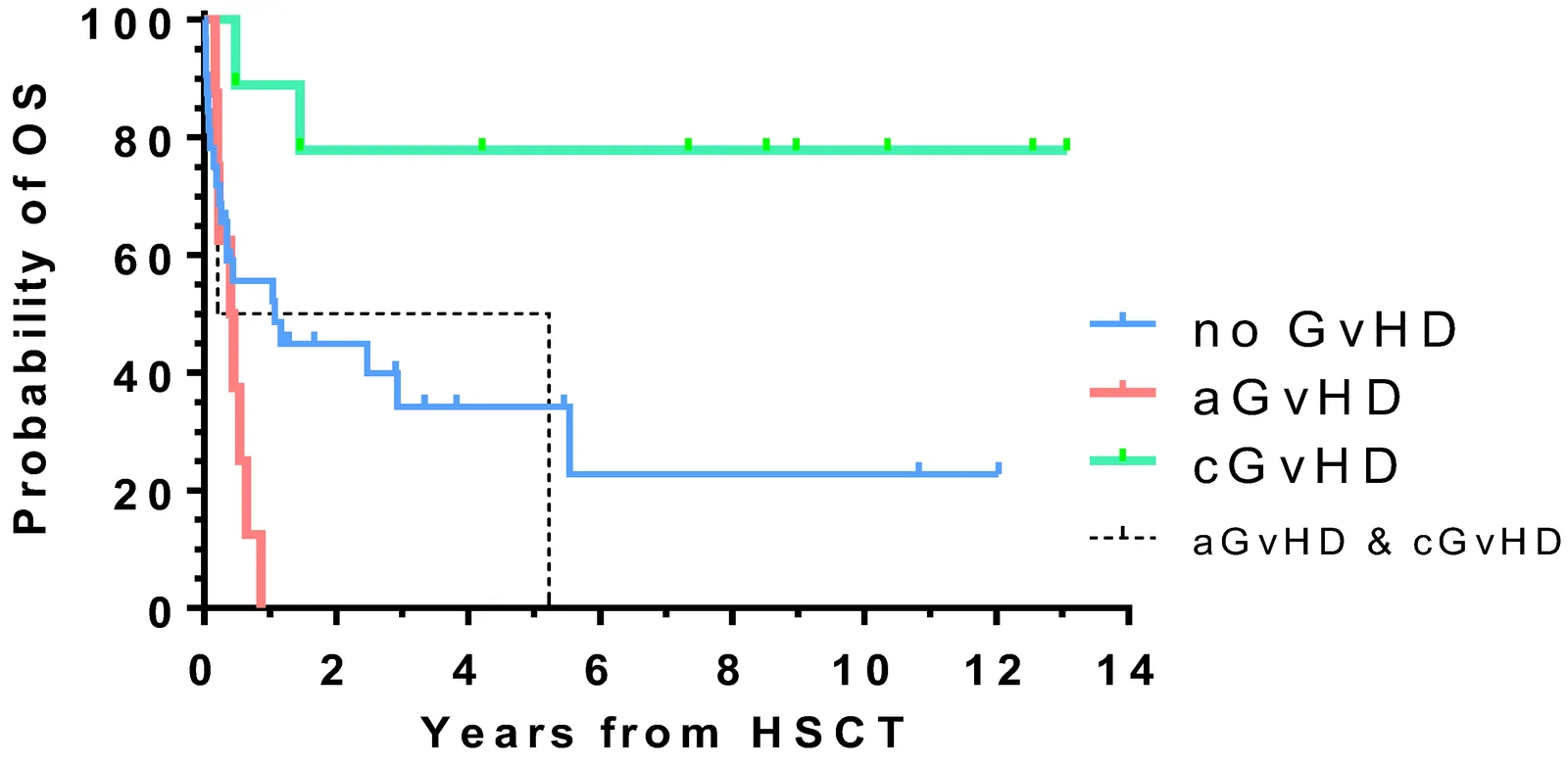
GvHD is of prognostic importance. GvHD was found to be an important prognostic variable. In patients with chronic GvHD survival was significantly improved compared to patients without GvHD or those with acute GvHD (aGvHD). Patients with aGvHD had the worst overall survival. In multivariable analysis including aGvHD, cGvHD and percentage of blasts in bm at transplantation, only cGvHD retained prognostic value. Abbreviations: aGvHD, acute graft-versus-host disease; cGvHD, chronic graft-versus-host disease; GvHD, graft-versus-host disease; HSCT, hematopoietic stem cell transplantation; OS, overall survival.

In our cohort of 51 HSCT patients, several clinical variables like age, type of disease, occurrence of acute or chronic GvHD, type of refractory disease (R versus RR) and percentage of bm blasts at transplantation were analyzed for their prognostic impact. There was no significant difference between the survival of patients with AML or those with ALL, or cases with R or RR disease. Similarly, age did not retain prognostic relevance (Table 4). The percentage of blasts in the bm at HSCT (<5%, 5–15%, >15%) showed a tendency toward significance (*p* = 0.089). aGvHD was an unfavorable prognostic factor for OS (*p* = 0.013) and RFS (*p* = 0.009) whereas cGvHD was associated with a favorable OS (*p* = 0.011) and RFS *p* = 0.034 (Figure 2). In a multivariable analysis including aGvHD, cGvHD and percentage of bm blasts at transplantation, only cGvHD remained a significant prognostic variable for OS (cGvHD *p* = 0.034; Table 4). Both aGvHD and cGvHD continued to serve as prognostic factors for RFS after assessment by uni- and multivariable analysis. In the AML cohort, the ELN-2009 classification was a significant prognostic factor for OS, both from the start of ClofCy treatment and from HSCT (*p* = 0.017 and *p* = 0.001, respectively; Figure S9a,b).

In addition, the effect of cGvHD was evaluated in a subgroup of 36 patients alive on day 100 after transplantation. The median survival of patients with cGVHD (*n* = 10) was not reached (5- and 10-year survival 80% and 68.6%, respectively) in contrast to the other patients (*n* = 26) with a median survival of 2.47 years (5- and 10-year survival 41.2% and 27.5%, respectively). Due to the low number of patients, the difference was not statistically significant (*p* = 0.052).

While cGvHD typically developed 90–180 days after transplantation, many patients died before day +100 and therefore did not develop cGvHD. To address this bias, we performed a landmark analysis including only patients alive at day +100 after HSCT. This analysis did not show a statistically significant OS difference between patients with and without cGvHD (*p* = 0.052, Figure S10). Nevertheless, median OS was not reached in patients with cGvHD compared with 2.5 years in those without cGvHD, and both 5- and 10-year survival rates were higher in the cGvHD group. To address a potential deviation from the proportional hazard assumption and immortal-time bias on an analysis of the prognostic value of aGvHD and cGvHD, we applied a Schoenfeld residuals test. Both aGvHD (*p* = 0.134) and cGvHD (*p* = 0.463) had no significant dependency on time and no interaction with time. In these analyses, 32 events were included in each model. The hazard ratio for aGvHD was 2.32 (95% confidence interval 1.01–5.35; *p* = 0.048) and 0.17 (95% confidence interval 0.039–0.74; *p* = 0.018) for cGvHD.

## 4. Discussion

In patients with R/RR acute leukemia, therapeutic options remain limited. HSCT is the only potentially curative treatment option for these patients. High blast cell counts and proliferative disease are frequently observed in these patients and can prevent HSCT or result in a poor outcome after HSCT [11,12,13,14]. In our cohort of patients with R/RR leukemia, 62.2% proceeded to HSCT following bridging with ClofCy for cytoreduction prior to HSCT. In these patients, long-term survival was achieved in a clinically meaningful proportion, with 5- and 10-year OS rates after transplantation of 38.4% and 31.0%, respectively.

In subgroup analyses, we also found that AML patients and ALL patients had an almost identical median OS from the start of ClofCy. This finding should be interpreted with caution, however, particularly given the relatively small number of patients with ALL compared to AML. In addition, ALL and AML both have a very poor prognosis in the refractory/relapsed setting. Moreover, there is a heterogeneity in the groups with regard to the number of prior treatment lines. Thus, disease stage, treatment history, response to ClofCy, and subsequent eligibility for HSCT may have contributed substantially to outcome and may have reduced the impact of type of disease on OS.

The findings highlight the potential of ClofCy as an effective debulking regimen that can serve for bridging to HSCT in this high-risk population. These data confirm our early results on the efficacy of this regimen [15]. Moreover, our data are in line with previous reports on other clofarabine-based regimens. Likewise, Locke et al. demonstrated the feasibility of clofarabine as cytoreductive therapy pre-HSCT with favorable survival outcomes [17]. Moreover, the efficacy of cytoreductive therapy prior to HSCT was also shown by Steckel et al. [18]. These authors explored sequential conditioning with high dose melphalan and showed that cytoreductive bridging strategies can yield meaningful survival in refractory AML [24]. Together, these data argue that strategies focusing on cytoreduction rather than CR prior to HSCT may be sufficient to allow timely transplant in patients with R/RR acute leukemia [15,17,18].

Repetitive cycles of traditional salvage strategies using high dose cytoreductive therapy often result in increased organ toxicity and prolonged periods of aplasia [2,16,17,18,19,20,21,22]. Karp et al. evaluated a combination of clofarabine and cyclophosphamide in patients with R/RR leukemias [24]. He showed that 20 mg/m^2^ of clofarabine together with 200–400 mg/m^2^ cyclophosphamide resulted in a higher percentage of treatment-related toxicity. The reduction in clofarabine to 10 mg/m^2^ improved the tolerability. In the light of this data, we applied clofarabine and cyclophosphamide (ClofCy) in our cohort at a dose of 10 mg/m^2^ and 200 mg/m^2^, respectively, and showed the feasibility of this regimen [15]. To evaluate the tolerability of this protocol, the hepatic and renal toxicities were evaluated in our study cohort. Therefore, organ function based on laboratory parameters, including AST, ALT, GGT, AP, bilirubin or Crea, were captured before therapy with ClofCy and on day 0 of HSCT. In patients that did not proceed to HSCT, these parameters were taken 30 days after the end of ClofCy substituting the data from day 0 of HSCT in the non-HSCT cohort. Overall, ClofCy-induced toxicity was moderate in most of our patients. Most hepatic and renal adverse events were grade 1–2 and not treatment-limiting. These results are in line with our prior published data [15] and the data of Locke et al. [11]. Severe grade 3–4 liver toxicity occurred in two patients only and a grade 3–4 increase occured in Crea in 5 patients. Thus, ClofCy appears to be less toxic compared to more intensive salvage regimens such as FLAG-Ida or FLAVIDA, both of which are associated with high rates of severe myelotoxicity and infectious complications [12,15,16]. Therefore, ClofCy may be particularly valuable in heavily pretreated patients with R/RR leukemias.

Severe infections were one of the most limiting factors in our study cohort. Indeed, infectious complications emerged not only as the leading cause of death but also as a major barrier to proceeding to HSCT. The high proportion of infection-related mortality, particularly among non-transplanted patients, underscores the vulnerability of this heavily pretreated population during cytoreductive therapy. These findings suggest that infections contribute both directly to early mortality and indirectly by precluding potentially curative transplantation. Consequently, strategies that achieve rapid disease control while minimizing prolonged neutropenia and infectious risk are critical to improving transplant eligibility and overall outcomes in patients with R/RR acute leukemia.

Prolonged exposure to multiple induction regimens heightens the risk of severe infections, organ dysfunction, and treatment-related mortality [20,21]. In our study, patients in the HSCT cohort had received a median of two chemotherapy regimens prior to initiation of ClofCy. In contrast, those who did not undergo transplantation because of a poor overall performance status and treatment-related complications received a median of three previous chemotherapy regimens prior to debulking. This observation suggests that restricting the number of intensive induction or salvage attempts prior to HSCT and incorporating ClofCy earlier may help maintain clinical stability, limit cumulative toxicity, and improve the likelihood of proceeding to HSCT. Considering efficacy in cytoreduction and moderate toxicity of ClofCy, an early application of this debulking strategy seems to be beneficial. Other variables including age, type of disease, and response to the debulking strategy were not of prognostic importance.

Several approaches have suggested that immediate HSCT following sequential conditioning is a feasible strategy to avoid chemotherapy-associated toxicity prior to transplantation [18,23,24,26]. Likewise, Stelljes et al. reported comparable overall survival (OS) and leukemia-free survival rates between patients receiving salvage chemotherapy before HSCT and those proceeding directly to HSCT [26,27]. However, due to proliferative disease, cytoreductive therapy was necessary to bridge 27% of patients in the study cohort to transplantation. We evaluated the efficacy of debulking with ClofCy as a strategy to bridge our patients to HSCT. Like in the ASAP trial by Stelljes et al., successful HSCT was feasible without achieving a complete remission (CR) prior to transplantation. Thus, ClofCy may provide a valuable middle ground by reducing disease burden and controlling leukemia without the toxicity associated with salvage chemotherapy [15,17,20,21].

Recently, targeted therapies and lower-intensity combination regimens have shown promising efficacy and tolerability. Gilteritinib has demonstrated clinical activity with a favorable safety profile in the relapsed/refractory setting in patients with FLT3 mutations [28,29]. Venetoclax in combination with azacitidine represents another treatment option, particularly for patients considered unfit for intensive chemotherapy [30]. Emerging data further suggest that venetoclax-based combinations may also be successfully used as bridging therapy prior to HSCT in selected patients with relapsed/refractory AML [31]. According to recent studies, menin inhibitors, including revumenib and ziftomenib, might represent potential treatment options for patients with KMT2A-rearranged or NPM1-mutated AML [32,33,34].

In our analysis, the 5-year OS of patients receiving ClofCy was 24%, which is lower than the 46% reported by Stelljes et al. [20,21]. Several differences in study design and patient characteristics may explain this discrepancy. Our study represents a retrospective analysis of consecutive patients without strict inclusion criteria, whereas the ASAP trial included only patients in first relapse or poor responders after the first induction cycle [26,27]. Accordingly, most patients in the ASAP trial had received only one prior therapy before inclusion (98.6% in the disease control arm and 95.0% in the remission induction arm) [26,27]. In contrast, patients in our cohort with induction failure had received at least two prior chemotherapy cycles, while patients with R/R disease had often undergone additional salvage therapies. As demonstrated in our analysis, a higher number of prior treatment cycles was associated with an unfavorable probability of proceeding to HSCT, which may partly explain the differences in outcome. Furthermore, the ASAP trial included AML patients only, whereas our cohort comprised both AML and ALL patients. Nevertheless, both studies emphasize that repeated attempts to achieve CR should be limited and that early HSCT after effective disease debulking should be pursued in patients with R/R leukemia. Our data suggest that ClofCy may represent a feasible debulking strategy in this setting.

As expected, overall survival (OS) in patients who underwent allogeneic HSCT was superior compared with the entire ClofCy-treated cohort. One- and three-year survival rates in these patients were 51% and 38%, respectively, with a long-term survival of 31% at 10 years. These results are comparable to those reported by Steckel et al. [18], who analyzed transplanted patients only and demonstrated 1- and 3-year survival rates of 46% and 34%, respectively. In the ASAP trial, which included patients with R/R AML, the 3-year OS after HSCT was approximately 55% in transplanted patients, compared with lower survival rates in patients who did not proceed to transplantation [26,27].

The prognostic impact of GvHD in our cohort is notable. Patients with cGvHD demonstrated superior OS and RFS, suggesting a sustained graft-versus-leukemia (GvL) effect [13,14]. In contrast, aGvHD was associated with inferior OS, highlighting the delicate balance between beneficial immune-mediated disease control and toxicity. The favorable association of cGvHD with survival in our cohort may reflect a sustained graft-versus-leukemia effect of donor-derived immune activity. Previous studies have reported lower relapse rates in patients developing cGvHD after allogeneic HSCT [12,13,14], although this association is not consistent across all disease groups. In contrast, aGvHD is associated with substantial treatment-related morbidity and mortality, which may explain its association with inferior survival in our cohort [35,36,37].

Several limitations of our study must be acknowledged. For example, the retrospective nature of the study, the relatively small patient numbers in subgroups, and the heterogeneity in conditioning regimens may limit the conclusions that can be drawn from our results. Moreover, our single-center experience may not fully reflect the patient populations and standard approaches in other centers.

## 5. Conclusions

In conclusion, ClofCy represents a promising debulking approach for patients with R/RR acute leukemias and can effectively bridge a substantial proportion of patients to HSCT. Early introduction of this regimen in R/RR patients may maximize transplant eligibility. The association of cGvHD with superior long-term survival highlights the importance of GvL effects in this high-risk setting. Future prospective studies are warranted to define the optimal use of ClofCy within the evolving therapeutic landscape of R/RR leukemias, particularly in comparison with immediate HSCT strategies and new targeted treatment approaches.

## Figures and Tables

**Table 1:** Patients’ characteristics.

Parameter	Median (Range)	Parameter	*n* (%)
All patients			
WBC; ×10^9^/L	4.4 (0.05–90.5)	HCT-CI	
ANC, ×10^9^/L	1.16 (0–35.8)	Low risk	40 (52.6%)
PLT, ×10^9^/L	36.5 (1–683)	Intermediate risk	25 (32.9%)
Hb, g/dl	9. 6 (5.7–14.7)	High risk	11 (14.5%)
Blasts in pb, %	9 (0–100)		
AML			
WBC; ×10^9^/L	4.2 (0.4–90.5)	HCT-CI	
ANC, ×10^9^/L	0.2 (0–10.56)	Low risk	34 (51.5%)
PLT, ×10^9^/L	35 (1–683)	Intermediate risk	22 (33.3)
Hb, g/dl	9.5 (6.2–14.7)	High risk	10 (15.5%)
Blasts in pb, %	10 (0–100)		
ALL			
WBC; ×10^9^/L	33.9 (0.1–0.9)	HCT-CI	
ANC, ×10^9^/L	0.14 (0.02–3.6)	Low risk	6 (66.7%)
PLT, ×10^9^/L	61 (7–410)	Intermediate risk	2 (22.2%)
Hb, g/dl	10.6 (5.7–13.4)	High risk	1 (11.1%)
Blasts in pb, %	1 (0–88)		

Abbreviations: ALL, acute lymphatic leukemia; AML, acute myeloid leukemia; ANC, all neutrophil count; Hb, hemoglobin; HCT-CI, the Hematopoietic Cell Transplantation-Comorbidity Index; pb, peripheral blood; PLT, platelets; WBC, white blood cell count.

**Table 2:** Effects of ClofCy on liver and renal function as assessed by CTCAE criteria.

Grade CTCEA	Improvement	Increase	
	1–2° *n* (%)	All *n* (%)	3–4° *n* (%)
AST	12 (14.8)	15 (18.5)	2 (2.4)
ALT	12 (14.8)	19 (23.4)	2 (2.4)
γ-GT	9 (11.1)	22 (27.1)	0 (0)
AP	9 (11.1)	11 (13.5)	0 (0)
Bilirubin	10 (12.5)	7 (8.7)	0 (0)
Creatinine	3 (3.7)	9 (11.1)	5 (6.1)

Abbreviations: ALT, alanine aminotransferase; AP, alkaline phosphatase; AST, aspartate aminotransferase; CTCAE, Common Terminology Criteria for Adverse Events; *n*, number of patients; γ-GT, gamma-glutamyltransferase.

**Table 3:** Comparison of HSCT and non-HSCT patients.

	HSCT	No HSCT	*p*-Value	
			Univariable	Multivariable
Number of chemotherapies prior to Clofcy, median	2	3	0.027	0.004
Age, median (IQR), years	44 (35–57)	49 (36–71)	0.214	0.261
R versus RR disease, *n*/*n*	23/28	11/20	0.392	0.137
Acute myeloid vs lymphoid disorder, *n*/*n*	42/9	24/7	0.585	0.377
HCT-CI			0.152	0.234
Low risk	27 (56.3%)	14 (48.3%)		
Intermediate risk	17 (35.4%)	8 (27.6%)		
High risk	4 (8.3%)	7 (24.1%)		
Blasts in bm after ClofCy			0.111	0.367
Blasts < 5%	26 (72.2%)	12 (63.2%)		
Blasts < 5–15%	4 (11.1%)	0 (0.0%)		
Blasts > 15%%	6 (16.7%)	7 (36.8%)		

Abbreviations: bm, bone marrow; HCT-CI, the Hematopoietic Cell Transplantation-Comorbidity Index; HSCT, hematopoietic stem cell transplantation; IQR, interquartile range; R, relapse; RR, relapsed refractory. The differences in univariable analysis for metric variables were assessed by Mann–Whitney and chi-square tests for categorical variables. Multivariable analysis was performed by nominal regression.

**Table 4:** Prognostic variable for overall survival.

	Overall Survival, *p*-Value		Relapse-Free Survival, *p*-Value	
	Univariable	Multivariable	Univariable	Multivariable
Age	0.289	-	0.264	-
Disease (AML versus ALL)	0.910	-	0.953	-
R versus RR disease, *n*/*n*	0.185	-	0.172	-
aGvHD	0.011	0.054	0.012	0.041
cGvHD	0.017	0.034	0.027	0.044
Blasts in bm at HSCT	0.083	0.226	0.096	0.226

Abbreviations: aGvHD, acute graft-versus-host disease; ALL, acute lymphozytic leukemia; AML, acute myeloid leukemia; cGVHD, chronic graft-versus-host disease; HSCT, hematopoietic stem cell transplantation; R, refractory; RR, relapsed refractory; *p*-values were assessed by uni- and multivariable Cox regression.

## Data Availability

Dataset is available on request from the authors.

## Supplementary Materials

The following supporting information can be downloaded at https://www.mdpi.com/article/10.3390/cancers18213593/s1. Table S1: Full-dose conditioning regimens used prior to HSCT; Table S2: Intermediate-dose conditioning regimens used prior to HSCT; Table S3: GvHD prophylaxis regimen; Table S4: Number of ClofCy cycles administered to all patients, HSCT and Non-HSCT-patients; Table S5: Changes in bone marrow blasts before and after treatment with ClofCy, Table S6: Changes in liver and renal parameters as assessed by CTCAE (grade 1–4) following therapy with ClofCy, in HSCT and non-HSCT patients; Table S7: Positive blood cultures with bacterial growth in aplasia following treatment with ClofCy, Table S8: Modifications of antimicrobial therapy in aplasia following treatment with ClofCy; Table S9: Early deaths* after ClofCy or HSCT; Figure S1: Patient selection and treatment pathways; Figure S2: Bone marrow blast percentage before and after therapy with ClofCy; Fisure S3: Maximum liver and renal toxicity during HSCT according to ClofCy cycles; Figure S4: Intravenous antimicrobial treatment during therapy with ClofCy; Figure S5: Overall Survival of all patients according to treatment period; Figure S6: Overall Survival of all Patients according to number of Clofcy cylces; Figure S7: Overall Survival of HSCT patients according to stem cell source; Figure S8: Overall survival of patients after HSCT according the Aalen–Johansen survival estimator and the Kaplan–Meier method; Figure S9: Survival of AML patients according the ELN-2009 after therapy with ClofCy or HSCT; Figure S10: Overall survival of patients with or without chronic GvHD.

## References

[1] Löwenberg B., Downing J.R., Burnett A. (1999). Acute myeloid leukemia. N. Engl. J. Med..

[2] Vellenga E., Griffin J.D. (1987). The biology of acute myeloblastic leukemia. Semin. Oncol..

[3] Faderl S., O’Brien S., Pui C.-H., Stock W., Wetzler M., Hoelzer D., Kantarjian H.M. (2010). Adult acute lymphoblastic leukemia: Concepts and strategies. Cancer.

[4] Brüggemann M., Raff T., Flohr T., Gökbuget N., Nakao M., Droese J., Lüschen S., Pott C., Ritgen M., Scheuring U. (2006). Clinical significance of minimal residual disease quantification in adult patients with standard-risk acute lymphoblastic leukemia. Blood.

[5] Grimwade D., Walker H., Oliver F., Wheatley K., Harrison C., Harrison G., Rees J., Hann I., Stevens R., Burnett A. (1998). The importance of diagnostic cytogenetics on outcome in AML. Blood.

[6] Grimwade D., Walker H., Harrison G., Oliver F., Chatters S., Harrison C.J., Wheatley K., Burnett A.K., Goldstone A.H., Medical Research Council Adult Leukaemia Working Party (2001). The predictive value of hierarchical cytogenetic classification in older adults with acute myeloid leukemia (AML): Analysis of 1065 patients entered into the United Kingdom Medical Research Council AML11 trial. Blood.

[7] Estey E.H. (2001). Prognostic factors in acute myelogenous leukemia. Leukemia.

[8] Collins-Underwood J.R., Mullighan C.G. (2010). Genomic profiling of high-risk acute lymphoblastic leukemia. Leukemia.

[9] Rowe J.M. (2010). Prognostic factors in adult acute lymphoblastic leukaemia. Br. J. Haematol..

[10] Bacher U., Kohlmann A., Haferlach T. (2010). Gene expression profiling for diagnosis and therapy in acute leukaemia and other haematologic malignancies. Cancer Treat. Rev..

[11] Locke F.L., Artz A., Rich E., Zhang Y., van Besien K., Stock W. (2010). Feasibility of clofarabine cytoreduction before allogeneic transplant conditioning for refractory AML. Bone Marrow Transplant..

[12] Craddock C., Labopin M., Pillai S., Finke J., Bunjes D., Greinix H., Ehninger G., Steckel N.-K., Zander A.R., Schwerdtfeger R. (2011). Factors predicting outcome after unrelated donor stem cell transplantation in primary refractory acute myeloid leukaemia. Leukemia.

[13] Doney K., Gooley T.A., Deeg H.J., Flowers M.E.D., Storb R., Appelbaum F.R. (2011). Allogeneic hematopoietic cell transplantation with full-intensity conditioning for adult acute lymphoblastic leukemia: Results from a single center, 1998–2006. Biol. Blood Marrow Transplant..

[14] Kebriaei P., Kline J., Stock W., Kasza K., Le Beau M.M., Larson R.A., van Besien K. (2005). Impact of disease burden at time of allogeneic stem cell transplantation in adults with acute myeloid leukemia and myelodysplastic syndromes. Bone Marrow Transplant..

[15] Rabitsch W., Böhm A., Bojic M., Schellongowski P., Wöhrer S., Sliwa T., Keil F., Worel N., Greinix H., Hauswirth A. (2014). Clofarabine/cyclophosphamide for debulking before stem cell transplantation. Eur. J. Clin. Investig..

[16] Zwitserloot A.M., Mavinkurve-Groothuis A.M.C., Galama J.M., Verweij P.E., Hoogerbrugge P.M., Warris A. (2012). Importance of neutropenia for development of invasive infections at various phases of treatment for hemato-oncological diseases in children. Scand. J. Infect. Dis..

[17] Locke F.L., Agarwal R., Kunnavakkam R., van Besien K., Larson R.A., Odenike O., Godley L.A., Liu H., Le Beau M.M., Gurbuxani S. (2013). A novel clofarabine bridge strategy facilitates allogeneic transplantation in patients with relapsed/refractory leukemia and high-risk myelodysplastic syndromes. Bone Marrow Transplant..

[18] Steckel N.K., Groth C., Mikesch J.-H., Trenschel R., Ottinger H., Kordelas L., Mueller-Tidow C., Schliemann C., Reicherts C., Albring J.C. (2018). High-dose melphalan-based sequential conditioning chemotherapy followed by allogeneic haematopoietic stem cell transplantation in adult patients with relapsed or refractory acute myeloid leukaemia. Br. J. Haematol..

[19] Ronnacker J., Urbahn M.A., Reicherts C., Kolloch L., Berning P., Sandmann S., Eßeling E., Call S., Floeth M., Marx J. (2024). Early blast clearance during sequential conditioning prior to allogeneic stem cell transplantation in patients with acute myeloid leukaemia. Br. J. Haematol..

[20] van Besien K., Artz A., Smith S., Cao D., Rich S., Godley L., Jones D., Del Cerro P., Bennett D., Casey B. (2005). Fludarabine, melphalan, and alemtuzumab conditioning in adults with standard-risk advanced acute myeloid leukemia and myelodysplastic syndrome. J. Clin. Oncol..

[21] Oliansky D.M., Appelbaum F., Cassileth P.A., Keating A., Kerr J., Nieto Y., Stewart S., Stone R.M., Tallman M.S., McCarthy P.L. (2008). The role of cytotoxic therapy with hematopoietic stem cell transplantation in the therapy of acute myelogenous leukemia in adults: An evidence-based review. Biol. Blood Marrow Transplant..

[22] Shahswar R., Beutel G., Gabdoulline R., Schwarzer A., Kloos A., Koenecke C., Stadler M., Gohring G., Behrens Y.L., Li Z. (2024). Fludarabine, cytarabine, and idarubicin with or without venetoclax in patients with relapsed/refractory acute myeloid leukemia. Haematologica.

[23] Schmid C., Schleuning M., Ledderose G., Tischer J., Kolb H.-J. (2005). Sequential regimen of chemotherapy, reduced-intensity conditioning for allogeneic stem-cell transplantation, and prophylactic donor lymphocyte transfusion in high-risk acute myeloid leukemia and myelodysplastic syndrome. J. Clin. Oncol..

[24] Karp J.E., Ricklis R.M., Balakrishnan K., Briel J., Greer J.M., Levis M.J., Gore S.D., Smith B.D., McDevitt M.A., Carraway H.E. (2007). A phase 1 clinical-laboratory study of clofarabine followed by cyclophosphamide for adults with refractory acute leukemias. Blood.

[25] Lee S.J., Williams K.M., Sarantopoulos S., Kitko C.L., Cutler C., Pidala J., Hill G.R., DeFilipp Z., Greinix H.T., Wolff D. (2025). NIH Chronic Graft-Versus-Host Disease Consensus Conference 2025 Update. Transplant. Cell Ther..

[26] Stelljes M., Middeke J.M., Bug G., Wagner-Drouet E.M., Müller L.P., Schmid C., Krause S.W., Bethge W., Jost E., Platzbecker U. (2024). Remission induction versus immediate allogeneic haematopoietic stem cell transplantation for patients with relapsed or poor responsive acute myeloid leukaemia (ASAP): A randomised, open-label, phase 3, non-inferiority trial. Lancet Haematol..

[27] Stelljes M., Middeke J.M., Bug G., Wagner-Drouet E.-M., Müller L.P., Schmid C., Krause S.W., Bethge W., Jost E., Platzbecker U. (2025). Disease risk but not remission status determines transplant outcomes in AML: Long-term outcomes of the ASAP trial. Blood.

[28] Perl A.E., Martinelli G., Cortes J.E., Neubauer A., Berman E., Paolini S., Montesinos P., Baer M.R., Larson R.A., Ustun C. (2022). Gilteritinib or Chemotherapy for Relapsed or Refractory FLT3-Mutated AML. N. Engl. J. Med..

[29] Cairoli R., Del Castello L., Imbergamo S., Pierdomenico E., Papayannidis C., Borlenghi E., Vetro C., Chiusolo P., Fumagalli M., Minotti C. (2025). Gilteritinib in FLT3-mutated acute myeloid leukemia: A real-world Italian experience. Cancer.

[30] Kantarjian H., Borthakur G., Daver N., DiNardo C.D., Issa G., Jabbour E., Kadia T., Sasaki K., Short N.J., Yilmaz M. (2024). Current status and research directions in acute myeloid leukemia. Blood Cancer J..

[31] Wei A.H., Loo S., Daver N. (2025). How I treat patients with AML using azacitidine and venetoclax. Blood.

[32] Arellano M.L., Thirman M.J., DiPersio J.F., Heiblig M., Stein E.M., Schuh A.C., Žučenka A., de Botton S., Grove C.S., Mannis G.N. (2025). Menin inhibition with revumenib for NPM1-mutated relapsed or refractory acute myeloid leukemia: The AUGMENT-101 study. Blood.

[33] Salman M.Y., Stein E.M. (2024). Revumenib for patients with acute leukemia: A new tool for differentiation therapy. Haematologica.

[34] Wang E.S., Issa G.C., Erba H.P., Altman J.K., Montesinos P., DeBotton S., Walter R.B., Pettit K., Savona M.R., Shah M.V. (2024). Ziftomenib in relapsed or refractory acute myeloid leukaemia (KOMET-001): A multicentre, open-label, multi-cohort, phase 1 trial. Lancet Oncol..

[35] Boyiadzis M., Arora M., Klein J.P., Hassebroek A., Hemmer M., Urbano-Ispizua A., Antin J.H., Bolwell B.J., Cahn J.-Y.Y., Cairo M.S. (2015). Impact of chronic graft-versus-host disease on late relapse and survival in 7489 patients after myeloablative allogeneic hematopoietic cell transplantation for leukemia. Clin. Cancer Res..

[36] Punatar S., Gupta A., Gawande J., Bagal B., Mathew L., Kannan S., Khattry N. (2016). Chronic graft versus host disease in acute leukemia patients undergoing allogeneic hematopoietic stem cell transplant: Analysis of risk factors, pattern and long term outcome. Indian J. Hematol. Blood Transfus..

[37] Horowitz M.M., Gale R.P., Sondel P.M., Goldman J.M., Kersey J., Kolb H.-J., Rimm A.A., Ringdén O., Rozman C., Speck B. (1990). Graft-versus-leukemia reactions after bone marrow transplantation. Blood.

