# Supplementary material for: Long-Term Survival of Patients with Relapsed/Refractory Acute Leukemia Receiving Clofarabine/Cyclophosphamide Prior to Hematopoietic Stem Cell Transplantation
Source: Cancers (Basel). 2026 Nov 9;18(21):3593. doi: 10.3390/cancers18213593 (PMC13762382; doi:10.3390/cancers18213593)
Supplement: Supplementary file 1 [file cancers-18-03593-s001.zip › cancers-4541383-supplementary.pdf]

Conditioning Regimens

Conditioning intensity and regimen choice were determined individually according to age, disease type, comorbidities, and attending physician preference. In younger and clinically fit patients, myeloablative regimens were favored, most frequently cyclophosphamide combined with high-dose total body irradiation (TBI 13.2). In patients of older age or with significant comorbidities, reduced-intensity or intermediate-dose conditioning was employed, with fludarabine-based regimens being most common. Other conditioning combinations (e.g., thiotepa/treosulfan/fludarabine) were used in selected cases, aiming to balance anti-leukemic activity with reduced non-hematologic toxicity. Details of the regimens administered in our cohort are summarized in Tables S1 and S2.

GvHD Prophylaxis

GvHD prophylaxis was tailored according to donor type, conditioning intensity, and clinical risk. Most patients received cyclosporine A (CSA) in combination with either methotrexate (MTX) or mycophenolate mofetil (MMF). ATG was added in mismatched or unrelated donor transplants in accordance with institutional protocols. Drug levels were closely monitored, and CSA target trough concentrations were typically maintained between 150–200 ng/mL. Table S3 provides an overview of prophylactic regimens used.

Supportive Care

All patients received comprehensive supportive care. Standard transfusion thresholds were set at hemoglobin <8 g/dL for red cell transfusion and PLT <10 or <20 × 10^9/L depending on bleeding risk. All patients received anti-infective prophylaxis with cotrimoxazole for Pneumocystis jirovecii from start of conditioning for HSCT, acyclovir for herpesvirus, and antifungal prophylaxis according to risk stratification. Antibacterial prophylaxis was considered in patients expected to experience prolonged neutropenia. Broad-spectrum antibiotics were initiated promptly in case of fever or suspected infection, and antifungal therapy was escalated based on clinical and radiographic criteria.

Supplementary Tables

Table S1. Full-dose conditioning regimens used prior to HSCT.

Regimen	n	Description
**Cy/TBI13.2**	26	Cyclophosphamide + total body irradiation 13.2 Gy
**Flu/TBI12**	4	Fludarabine + total body irradiation 12 Gy
**Thio5Treo30Flu**	2	Thiotepa 5 mg/kg, treosulfan 30 g/m², fludarabine
**Thio10Treo90Flu**	4	Thiotepa 10 mg/kg, treosulfan 90 g/m², fludarabine
**FCM-ATG**	2	Fludarabine + cyclophosphamide + melphalan + ATG
**Thio10/Treo30**	1	Thiotepa 10 mg/kg + treosulfan 30 g/m²

Abbreviations: ATG, anti-thymocyte globulin; Gy, Grey.

Table S2. Intermediate-dose conditioning regimens used prior to HSCT.

Regimen	n	Description
**FLAMSA**	6	Fludarabine + amsacrine + cytarabine + cyclophosphamide
**TB2F**	2	Fludarabine + total body irradiation 2 Gy
**FluTreo30**	2	Fludarabine + treosulfan 30 g/m²
**FB4**	1	Fludarabine + busulfan 4 mg/kg
**Unknown protocol**	1	Applied regimen not recorded

Abbreviations: Gy, Grey.

Table S3. GvHD prophylaxis regimens.

Prophylaxis	n	Notes
**CSA + MTX**	23	Seattle protocol (CSA target 150–200 ng/mL)
**CSA + MMF**	26	MMF 15 mg/kg/day from day 0
**ATG**	2	–
**CSA + MMF + ATG**	16	Subgroup of CSA+MMF patients
**CSA + MTX + ATG**	1	–

Abbreviations: ATG, anti-thymocyte globulin; CSA, cyclosporine A; MMF, mycophenolate mofetil; MTX, methotrexat.

Table S4. Number of ClofCy cycles administered to all patients, HSCT and Non-HSCT-patients.

Cycles	All patients	HSCT	No HSCT
**1**	23	13	10
**2**	27	18	9
**3**	20	14	6
**4**	11	6	5
**5**	1	0	1
**Median (range)**	**2 (1–5)**	**2 (1–4)**	**2 (1–5)**

Abbreviations: ClofCy, clofarabine/cyclophosphamide; HSCT, hematopoietic stem cell transplantation.

Table S5. Changes of bone marrow blasts before and after treatment with ClofCy.

n	all	HSCT	Non-HSCT
**no change**	7	4	3
**reduction**	36	23	12
**increase**	5	3	2
	49	30	17
%			
**no change**	14,6	13.3	17.6
**reduction**	75.0	76.7	70.6
**increase**	10.4	10.0	11.8
	100.0	100.0	100.0

Abbreviations: HSCT, hematopoietic stem cell transplantation; ClofCy, clofarabine/cyclophosphamide; n, number of patients.

Table S6. Changes in liver and renal parameters as assessed by CTCAE (grade 1-4) following therapy with ClofCy, in HSCT and non-HSCT patients.

	**No HSCT**			**HSCT**		
	Improvement	Worsening		Improvement	Worsening	
	**1-2°**	**all**	**3-4°**	**1-2°**	**all**	**3-4°**
	**n (%)**	**n (%)**	**n (%)**	**n (%)**	**n (%)**	**n (%)**
AST	4 (12.9)	8 (25.8)	2 (6.4)	8 (16)	7 (14)	0 (0)
ALT	3 (9.6)	8 (25.8)	2 (6.4)	9 (18)	11 (22)	0 (0)
γ GT	4 (12.9)	11 (35.4)	0 (0)	7 (14)	11 (22)	0 (0)
AP	4 (12.9)	4 (12.9)	0 (0)	5 (10)	7 (14)	0 (0)
Bilirubin	5 (16.1)	4 (12.9)	0 (0)	5 (10.2)	3 (6.1)	0 (0)
Creatinine	1 (3.2)	8 (25.8)	5 (16.1)	2 (4)	1 (2)	0 (0)

Abbreviations: ALT, alanine aminotransferase; AP, alkaline phosphatase; AST, aspartate aminotransferase; CTCAE, Common Terminology Criteria for Adverse Events; γ-GT, gamma-glutamyltransferase.

Table S7. Positive blood cultures with bacterial growth in aplasia following treatment with ClofCy.

Number of positive cultures	0	1	2	3	4
**HSCT, n (%)**	34 (65.4%)	13 (25%)	4 (7.7%)	0 (0%)	**1 (1.9%)**
**Non-HSCT, n (%)**	11 (36.7%)	7 (23.3%)	7 (23.3%)	4 (13.3%)	**1 (3.3%)**
**All patients, n (%)**	45 (54.9%)	20 (24.4%)	11 (13.4%)	4 (4.9%)	**2 (2.4%)**

Abbreviations: HSCT, haematopoietic stem cell transplantation; n, number of patients. p=0.009, as assessed by Chi square test.

Table S8. Modifications of antimicrobial therapy in aplasia following treatment with ClofCy.

Modifications of Therapy	0	1	2	>2
**HSCT, n (%)**	13 (28.9%)	10 (22.2%)	12 (26.7%)	10 (22.2%)
**Non-HSCT, n (%)**	4 (16.7%)	2 (8.3%)	3 (12.5%)	15 (62.5%)
**All patients, n (%)**	17 (24.6%)	12 (17.4%)	15 (21.7%)	25 (36.2%)

Abbreviations: AM, antimicrobial; HSCT, haematopoietic stem cell transplantation; n, number. p=0.011, as assessed by Chi square test.

Table S9. Early deaths* after ClofCy or HSCT.

Cause of death	All patients (n)	Non-HSCT (n)	Non-HSCT – no day 0 (%)	HSCT (n)	Median age (range)
**Sepsis / MOF**	21	16	14 (87.5%)	5	47.5 (26–62)
**Clinical deterioration**	4	4	4 (100%)	0	62.5 (34–65)
**Refractory disease**	5	4	3 (75.0%)	1	39.5 (22–53)
**Aspergillus pneumonia**	2	2	2 (100%)	0	57.5 (54–61)
**Brain bleeding**	3	3	3 (100%)	0	63 (54–64)
**aGvHD**	2	0	–	1	–
**Candida infection**	1	1	1 (100%)	0	35
**COVID-19 infection**	1	1	1 (100%)	0	43

Abbreviations: aGvHD, acute graft-versus-host disease; ClofCy, clofarabine plus cyclophosphamide; COVID-19, coronavirus disease 2019; HSCT, haematopoietic stem cell transplantation; MOF, multiorgan failure. * Early death was defined as within 60 days after therapy with Clofcy or day 0 in case of HSCT patients.

Supplementary Figures

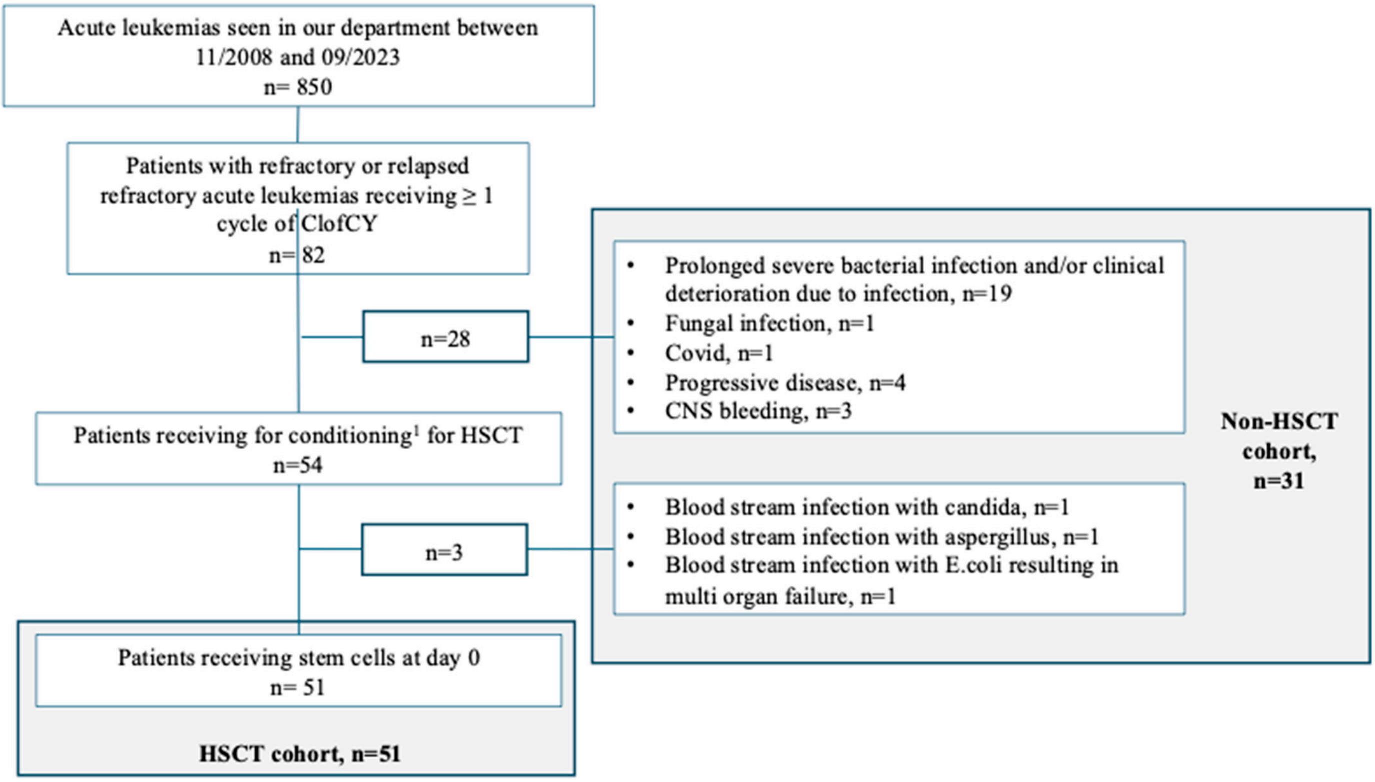

Figure S1. Patient selection and treatment pathways.Patient selection and treatment pathway of patients receiving ClofCy. The flowchart shows the selection of patients with relapsed/refractory acute leukemia, subsequent conditioning for HSCT, and reasons for not proceeding to HSCT. Abbreviations: ClofCy, Chemotherapy applying clofarabine (10 mg/m^2) and cyclophosphamide (200 mg/m^2); CNS, central nervous system; HSCT, hematopoietic stem cell transplantation; n, number.[1] A detailed list of the full- and intermediate-dose conditioning regimens is provided in Tables S1 and S2.

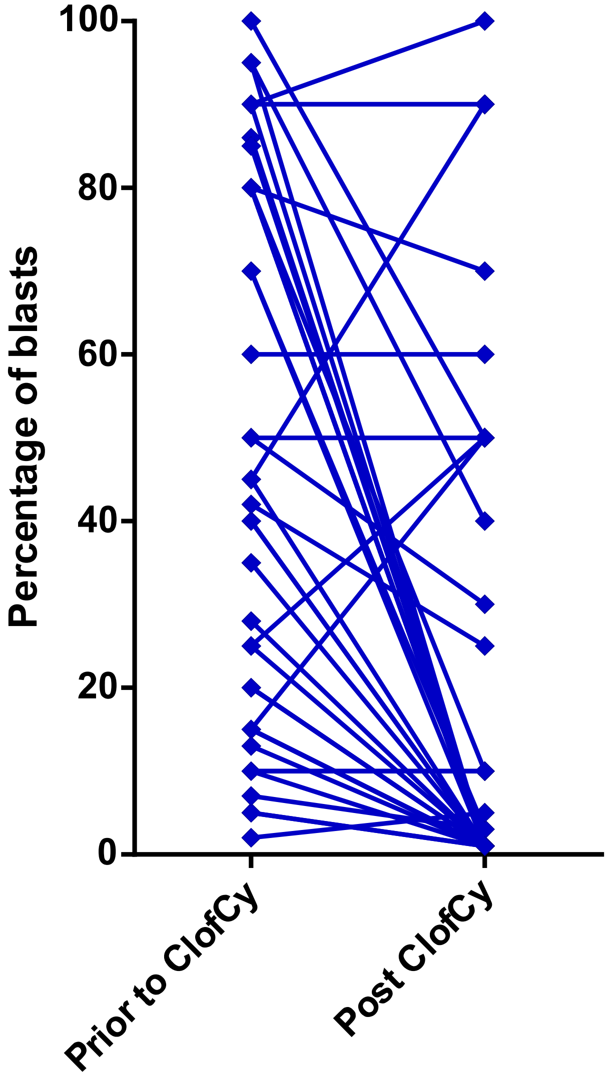

Figure S2. Bone marrow blast percentage before and after therapy with ClofCy. Figure S2 shows changes in bone marrow blast percentage before and after therapy with ClofCy. Abbreviations: ClofCy, clofarabine/cyclophosphamide.

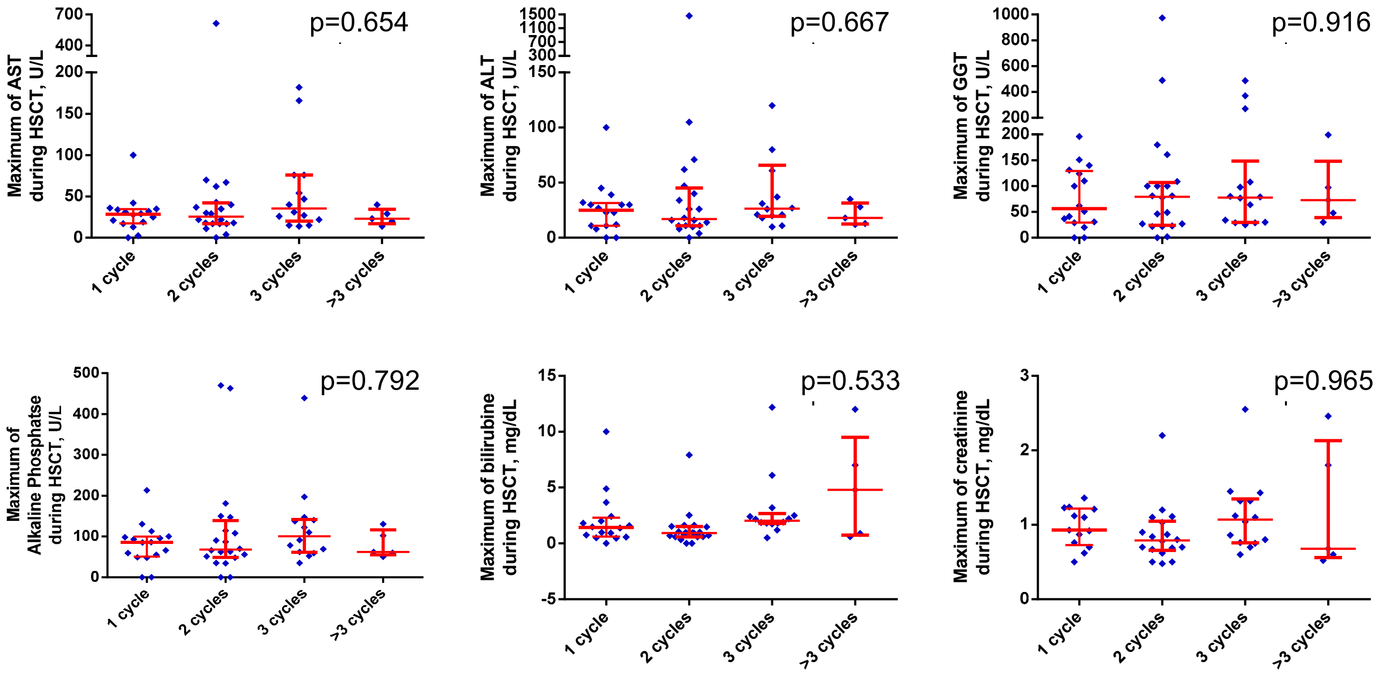

Figure S3. Maximum liver and renal toxicity during HSCT according to ClofCy cycles. Figure S3 shows medians and interquartile ranges for a comparison of maximum toxicity with regard to liver and renal function during HSCT according to ClofCy cycles received. No significant difference was observed between patients who received one, two, three, or four-five cycles of Clofcy; the analysis was performed using the Jonkheere-Terpstra test. Abbreviations: ALP, alkaline phosphatase; ALT, alanine aminotransferase; AST, aspartate aminotransferase; bili, bilirubin; ClofCy, clofarabine/cyclophosphamide; crea, creatinine; GGT, gamma-glutamyl transferase; HSCT, hematopoietic stem cell transplantation.

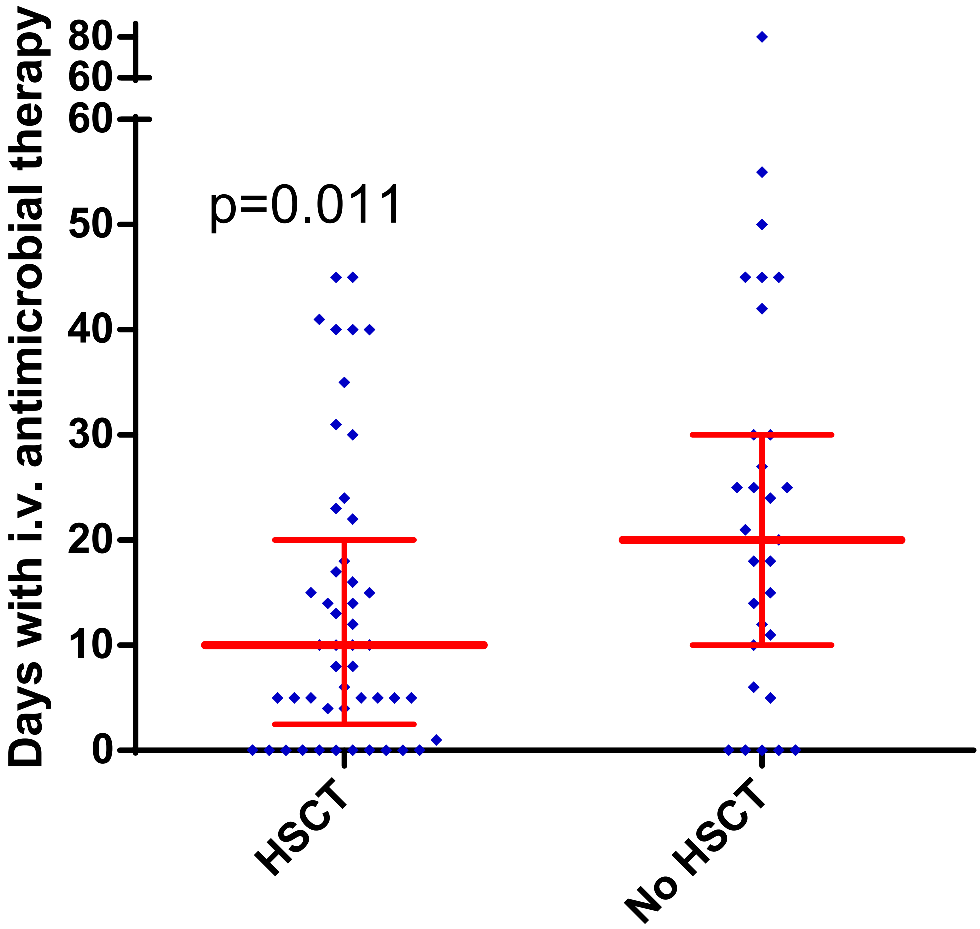

Figure S4. Intravenous antimicrobial treatment during therapy with ClofCy**.** Figure S4 depicts median and interquartile range for duration of intravenous antimicrobial treatment during therapy with ClofCy and before eventually proceeding to HSCT. In the HSCT cohort the requirement of intravenous antimicrobial therapy was significantly lower (p=0.011) as compared to the non-HSCT cohort. Abbreviations: ClofCy, clofarabine/cyclophosphamide; HSCT, hematopoietic stem cell transplantation; i.v., inter venous.

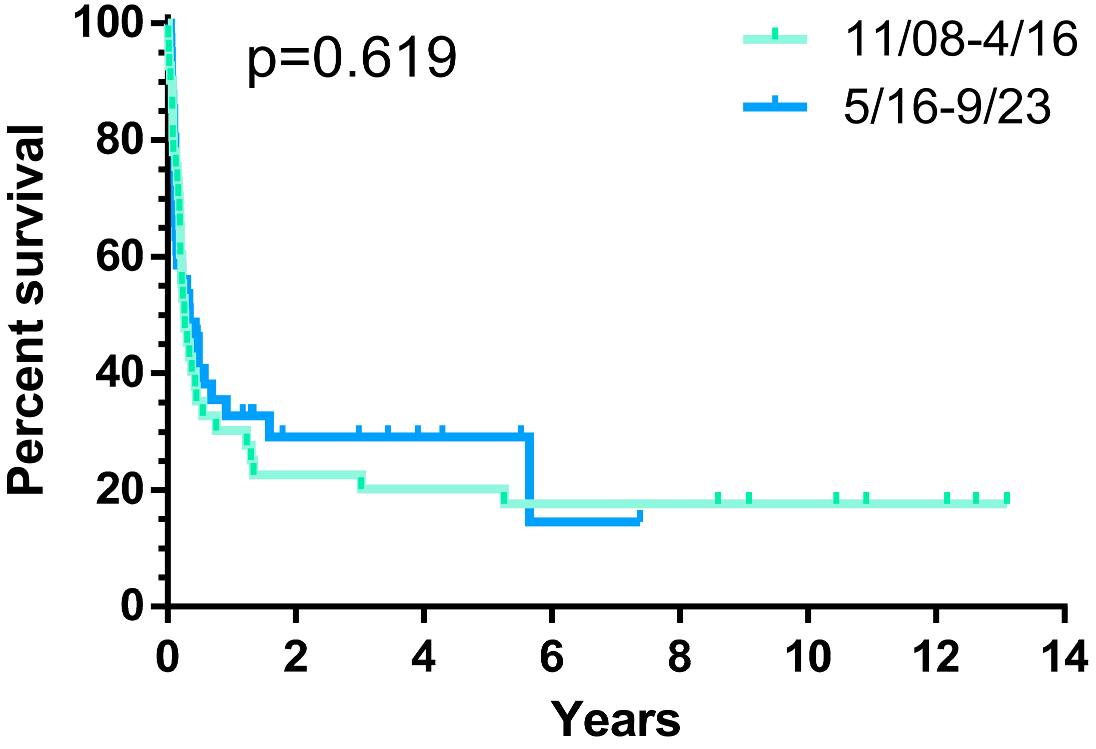

Figure S5. Kaplan-Meier estimates of overall survival of all patients according to treatment period. Figure S5 depicts Overall Survival of all patients according to treatment period. There was no significant difference in overall survival with regard to treatment period (11/08-03/15 vs. 03/15-09/23, p=0.917).

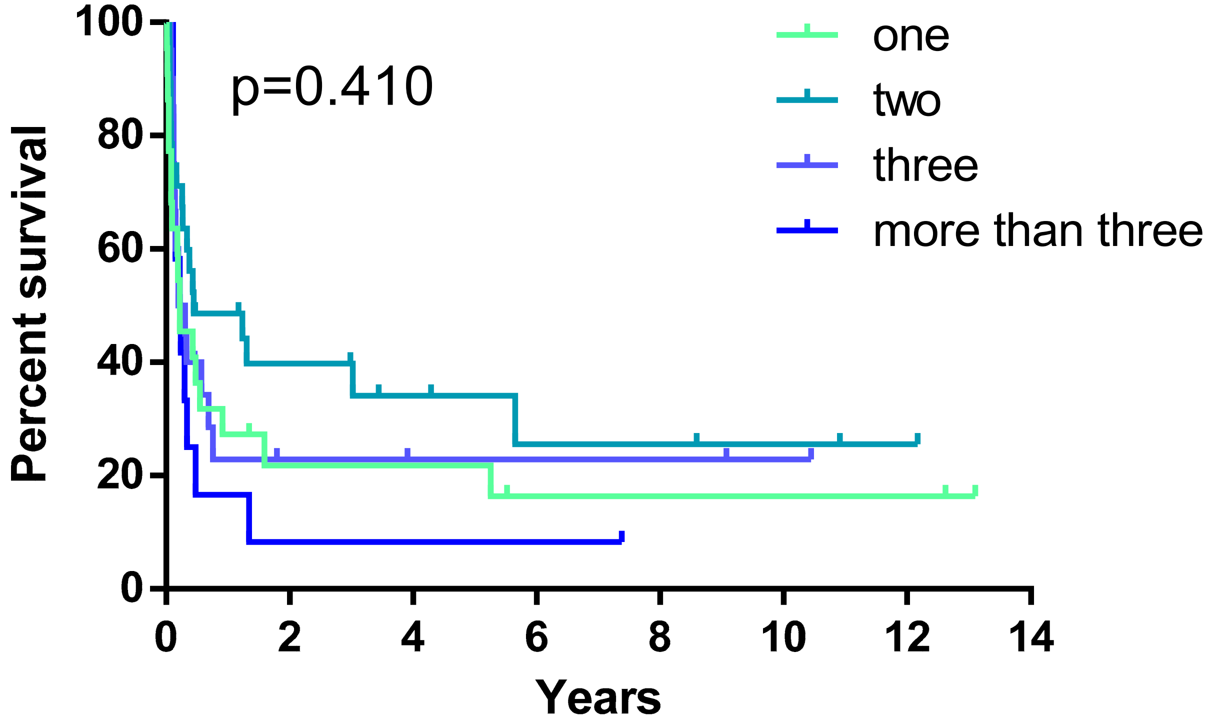

Figure S6. Kaplan-Meier estimates of overall survival of all patients according to number of Clofcy Cyles. Overall Survival of all patients with regard to number of Clofcy cycles applied. There was no significant difference when comparing 1 cycle; 2 cycles; 3 cycles; >3 cycles (p=0.41). Abbreviations: ClofCy, clofarabine/cyclophosphamide.

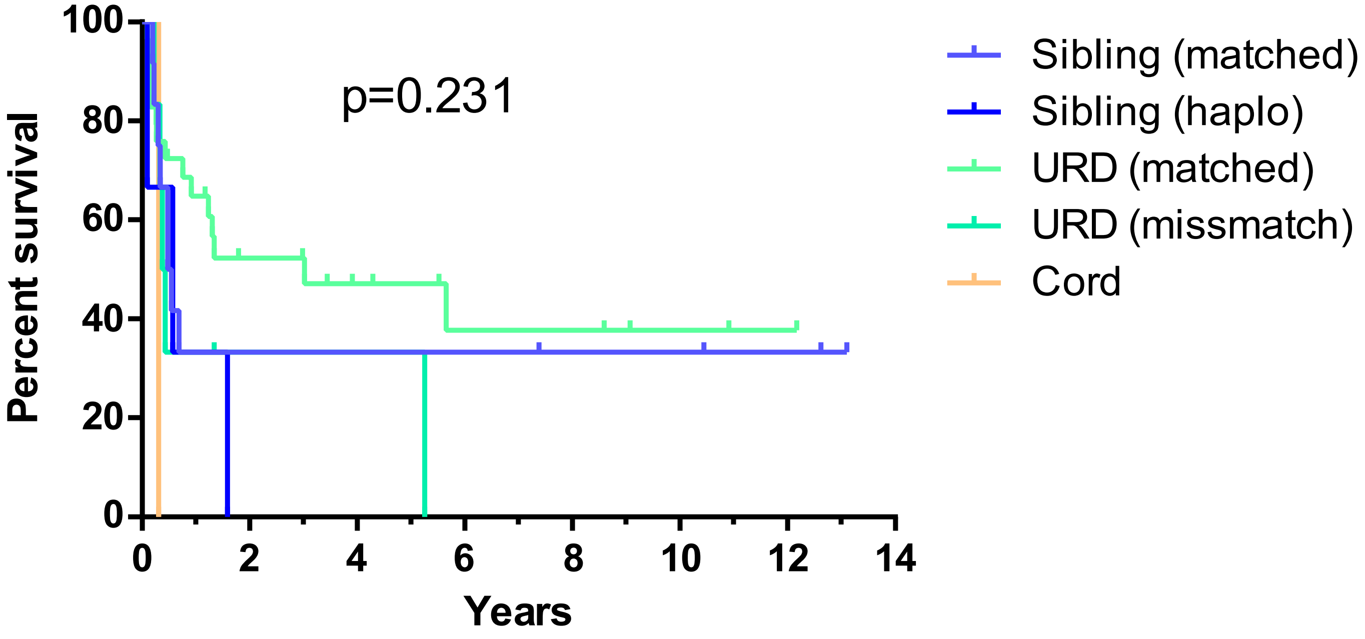

Figure S7. Kaplan-Meier estimates of overall survival of HSCT patients according to stem cell source. Overall Survival of HSCT patients according to source of stem cells. There was no significant difference in overall survival when comparing matched sibling, haploidentical sibling, matched unrelated, mismatched unrelated donor or cord blood (p=0.231). Abbreviations: Sibling (matched), matched sibling donor; Sibling (haplo), haploidentical sibling donor; URD (matched), matched unrelated donor; URD (mismatch), mismatched unrelated donor; Cord, umbilical cord blood transpantatin.

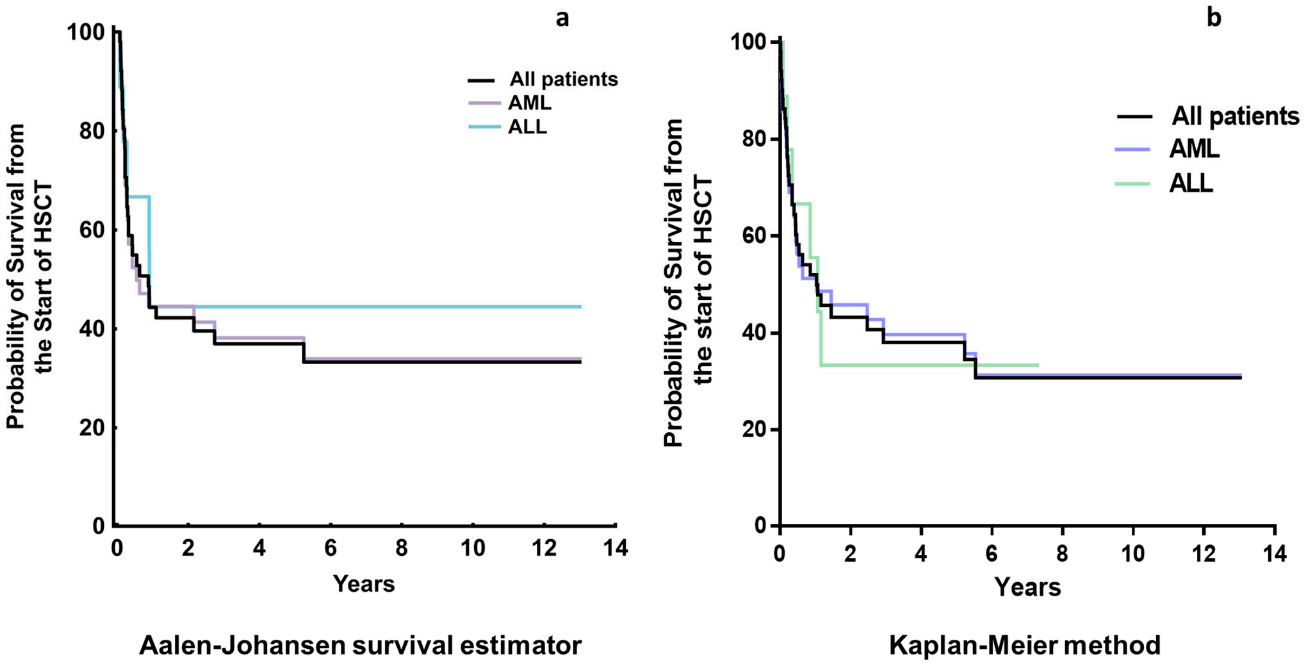

Figure S8. Overall survival of patients after HSCT according the Aalen-Johansen survival estimator and the Kaplan-Meier method. To address selection and immortal-time bias in the comparison between HSCT and non-HSCT patients, we have calculated the survival of our patients according the Aalen-Johansen survival estimator (a). This considered that patients must survive and remain clinically eligible long enough to undergo HSCT, whereas early deaths and severe infections are inherently more likely to occur in the non-HSCT group. As visible shown in fin the graph above, no major difference was found compared to the survival calculated according the Kaplan-Meier method (b).

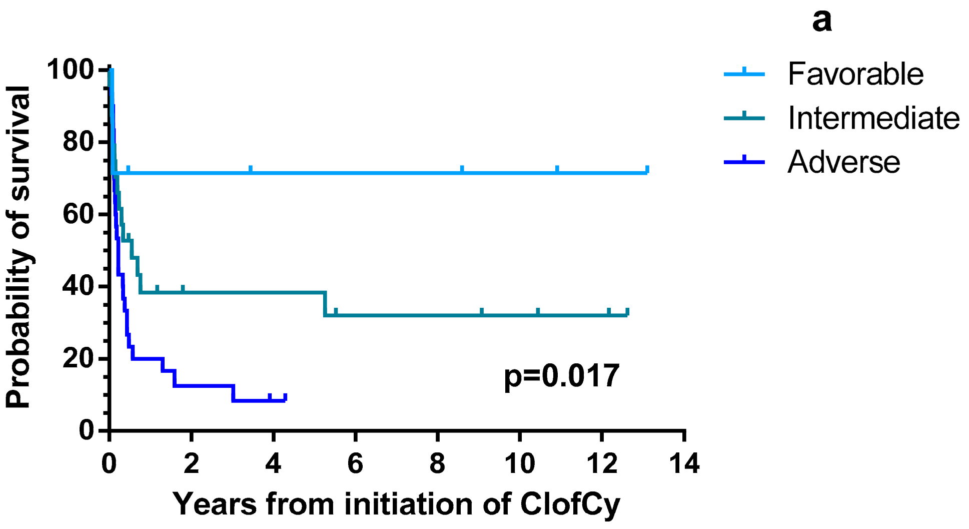

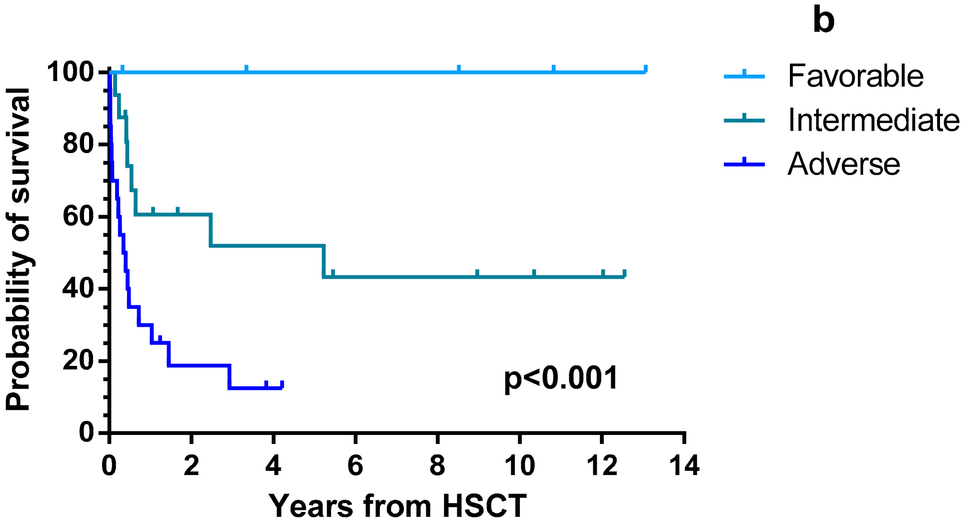

Figure S9. Kaplan-Meier estimates of survival of AML patients according the ELN-2009 after therapy with ClofCy or HSCT. Survival of AML patients according the ELN-2009 classification was calculated with the method of Kaplan and Meier. A significant difference in the survival from day one of ClofCy (a; $p=0.017$) and day one of HSCT (b; $p<0.001$) was found between patients in the favorable, intermediate and adverse risk groups.

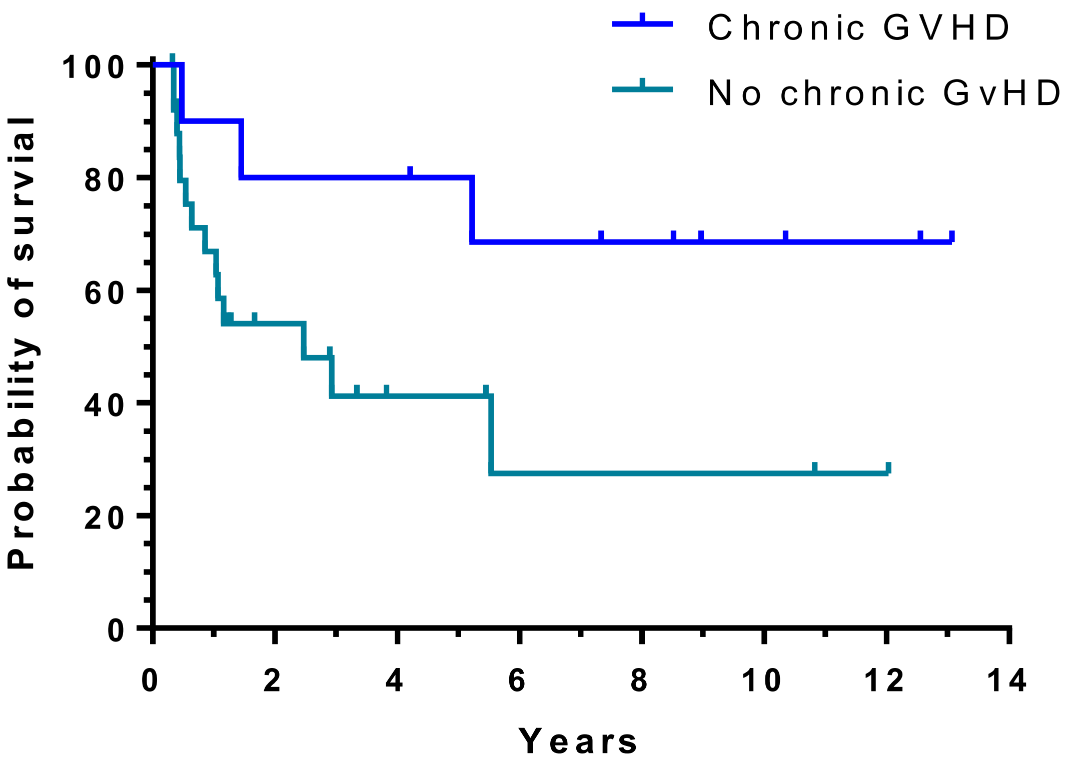

Figure S10. Kaplan-Meier estimates of overall survival of patients with or without chronic GvHD. In this analysis the survival of patients with or without chronic GvHD (cGvHD) was compared. The minimize a bias due to acute transplant related mortality a Landmark analysis using day +100 after HSCT as the cutoff was done. Patients that developed cGvHD had an improved survival. Abbreviation: GCHD, graft-versus-host disease. Abbreviations: ClofCy, clofarabine/cyclophosphamide; HSCT, hematopoietic stem cell transplantation.
